# Hidden Populations for Healthcare Financial Protection in the Super-Aging Society: Closing the Gap Between Policy and Practice

**DOI:** 10.1007/s10615-023-00914-x

**Published:** 2024-01-16

**Authors:** Noriko Sasaki, Megumi Rosenberg, Jung-ho Shin, Susumu Kunisawa, Yuichi Imanaka

**Affiliations:** 1 Department of Healthcare Economics and Quality Management, Graduate School of Medicine, Kyoto University, Yoshida Konoe-cho, Sakyo-ku, Kyoto, 606-8501 Japan; 2 World Health Organization Centre for Health Development (WHO Kobe Centre - WKC), 1-5-1 Wakinohama-Kaigandori, Chuo-ku, Kobe, 651-0073 Japan

**Keywords:** Financial protection, Social work, Health equity, Super-aging society, Universal health coverage

## Abstract

Despite the remarkable health achievements of Japan’s universal health coverage since 1961, along with numerous social programs to ensure financial protection, a growing proportion of the older population reportedly experiences financial hardship for essential health care. The socio-behavioral and economic situation of the households in need and the effective policy interventions remain unknown. To identify the reasons behind older persons’ financial hardship and the effective policy interventions, we performed a questionnaire survey of social workers in all hospitals, local government offices and social service agencies across six prefectures in Kansai region. Data from 553 respondents revealed that the financial difficulties related to health care are often closely intertwined with social and mental health hardships experienced by older people and their families. Notably, potentially helpful programs including ‘free/low-cost medical treatment program’ and the adult guardianship system for dementia were infrequently used. Moreover, male, social workers at local offices/agencies, and less than 10 years’ professional experience associated with infrequent use of key protective programs. To close the gap between policy and practice, policies should focus on clients’ daily living needs, and new frontline social workers should receive lifelong training that incorporates their own backgrounds, experiences, and values, including the use of anti-oppressive gerontological approaches.

**Supplementary Information:**

The online version contains supplementary material available at 10.1007/s10615-023-00914-x.

## Introduction

Universal health coverage (UHC) must protect citizens against catastrophic costs that may drive them into poverty (World Health Organization “WHO”, 2019a; WHO, 2018; Tomita, 2017; WHO and International Bank for Reconstruction and Development “IBRD”/The World Bank, 2021). Catastrophic health spending and levels of unmet need are high and rising in many countries, especially among poorer households (WHO, 2019a) due to economic stagnation, growing economic inequality, the changing course of diseases, and aging populations. Financial hardship is caused by out-of-pocket payments, and lack of financial protection may lead to deepen poverty, exacerbate health and socioeconomic inequalities (WHO, 2019a). When focusing on age, and considering the unit of analysis as the household, people living in older households which include at least one older person aged 60 + as well as those living in multigenerational households which include adults (20 to 59 years) are reported to face the highest incidence/rates of catastrophic/impoverishing health spending, using the data based on a sample of 92 countries across all UN regions except North America and Oceania (WHO and IBRD/The World Bank, 2021).

Japan, the world’s leading “super-aging” society (Ikegami, 2014; D’Ambrogio, 2020) with 29% of the population who are aged 65 or older (D’Ambrogio, 2020; WHO and IBRD/The World Bank, 2021), faces multiple challenges in sustaining its social security system. Despite the remarkable success in health since Japan achieved UHC in 1961, along with social welfare system and multiple public programs to ensure financial protection, a growing proportion of the older population is reported to experience financial hardship for essential health care (D’Ambrogio, 2020; Cabinet Office, Government of Japan, 2021). In 2022, more than 30% of the population felt somewhat anxious about their daily expenses; mean annual household income of this age group (3.1million Japanese Yen) was almost half that of households in which everyone was younger than 65, approximately 5.5 million Japanese Yen, (Cabinet Office, Government of Japan, 2021). In addition, over half of households that receive public assistance include 65 + years people, and the proportion has been increasing since 2015 (Ministry of Health, Labour and Welfare “MHLW”, 2021a; Sakamoto et al., 2018). Lower-income and older subscribers in Japan’s National Health Insurance (NHI) often cannot afford the high premium rate, and many lose insurance coverage after failing to pay their premiums for several months. Those who cannot afford to make their insurance payments and do not meet the criteria for public assistance are defined as borderline poor. Borderline poor and even poor people who meet the criteria may not use essential health care services because the out-of-pocket expenses are too high (Health & Global Policy Institute, 2009; Sakamoto et al., 2018).

The exact number of poor and borderline poor people who are being left behind is still unknown, despite the range of existing policies and public programs designed to support them including high-cost medical expense benefits (reimbursements above a set monthly threshold), publicly funded low-cost medical treatment, and public assistance or welfare (Komatsu, 2018; Yoshinaga et al., 2019). Benefits of these policies and programs may be offset by rising levels of informal employment, decreasing wages, and an increase in the number of older low-income pensioners that creates low-income households (Komatsu, 2018; Nishigaki, 2011; Sato, 2014). Their situation could be worsened by unexpected natural disasters, poorer health, and sometimes abusive family relationships (Komatsu, 2018; Nishigaki, 2011; Sato, 2014).

Since the national Long-Term Care Insurance (LTCI) system was introduced in 2000 (Ikegami, 2014; Health, Labour and Welfare Statistics Association, 2021a; 2021b), various services have become available for older people to reduce the caregiving burden on their family members including the financial burden. In addition to certified social workers, other related staff, including community social workers and local government staff, have been engaged in coordinating these services in Japanese settings (Sakamoto et al., 2018). In this regard, these social work professionals are the primary care coordination gatekeepers who have an accurate understanding of the challenges faced by the older population (Baines, 2017). On the other hand, from the service user’s perspective, LTCI services and other protective policies and programs have been fragmented, too complicated to understand, and difficult to access (Komatsu, 2018; Nishigaki, 2011). In particular, older people with cognitive impairment or dementia, found it harder to receive the services they needed (Nishigaki, 2011; Sato, 2014).

In general, individuals must apply to the local government to access health care services or financial support. Applicants need to be familiar with the health system or must rely on the support of social welfare professionals to assist with the application process. The complexity of the system is said to be one of the primary obstacles preventing older people from utilising appropriate services (Inaba et al, 2020; Iwasaki et al, 2014; Nishigaki, 2011). Some local governments are trying to construct a seamless system to tackle this challenge such as in Yasu city in Shiga prefecture (Sano et al, 2023). Here, an integrated network monitors safety for the citizens as consumers. It shares information about who is in need of protection with various organizations, such as public health centers, clinics, police, financial institutions, retail stores, and civil societies (Sano et al, 2023). These activities could be a prototype for improving health service accessibility of clients.

However, individuals from diverse socioeconomic backgrounds have had difficulties in making informed decisions and face additional hurdles to receiving the services they need (Iwasaki et al, 2014; Sano et al, 2023). The adult guardianship system established in 2000, which provides legal support to those without sufficient capacity to make decisions because of mental or intellectual disabilities or dementia, is expected to alleviate these sorts of challenges, but the uptake has been slow and the system has its own problems (Ministry of Justice, 2023; Sano et al, 2023). Negative attitudes of some local government staff, such as discouraging citizens from properly applying, have also been reported to hinder the seamlessness of the application process (Sato, 2014; Yamaguchi, 2020; Inaba et al., 2020; NPO Japan Medical Social Work Group, 2021). However, the factors which are related to the negative attitudes of the social work professionals are understudied (Japanese Government Statistics, 2017; Matsuda, 2016).

Taking the multiple standpoints of stakeholders into account, (Michie et al., 2005; Michie et al., 2011; Michie et al., 2014; Nilsen et al., 2010) the aim of this study is twofold. The first aim of this study is to critically examine the difficulties currently faced by older population in paying for healthcare services. This aim will be examined from the perspective of two types of social workers: that of medical social workers who work at hospitals (hereinafter, “MedSWs”), and that of social workers who work in the local government, Social Welfare Council, or community general support center (hereinafter, “SWs”). By integrating the results from these two perspectives, we expect to identify broader needs for health services related to the daily lives of older people, including the socio-behavioral and economic situation of households. The second aim is to explore the social worker’s personal factors associated with the underused policies and programs of financial protection, clarifying the service provider-level element which may hinder or facilitate the program use.

## Methods

### Data Collection

#### Questionnaire Survey

Two types of a questionnaire, one for MedSWs and another for SWs, were developed based on the following three steps: (i) a literature review, (ii) the creation of a pilot questionnaire and (iii) the elaboration of the questionnaire through semi-structured, face-to-face interviews with five conveniently selected certified social workers working in two hospitals and one community general support center (See Appendix A). Regarding community general support centers, they were established in 2004 under the responsibility of local municipalities, who are the insurers of the NHI, to provide community-based integrated care especially for the growing number of older people living in the community with the need for health and long-term care. For the literature review, information was gathered from literature, reports on national policies and programs (laws and guidelines), policy review documents such as white papers, and peer-reviewed articles. The constructs of interest in the questionnaire were identified based on a broad review of the current financial protection systems available to older people and their households in Japan. To help ensure the face validity of the survey, the questionnaire was pilot tested, and repeatedly checked through meetings and emails.

Paper copies of the questionnaires were distributed by mail to all the hospitals (n = 1,121), community general support centers (n = 854), social welfare councils (n = 248), and local governments (n = 249) in the Kansai region of all six prefectures and their municipalities, from October to November 2021. We identified all hospitals in the Kansai region using the openly available list of Administrative Reports on Hospital Bed Function by Ward Matching Technique (https://www.mhlw.go.jp/stf/seisakunitsuite/bunya/0000055891.html), excluding hospitals specified for maternity/pediatrics. Social workers of all the local governments and community health care agencies were also identified using publicly available address list, including all six prefectures and 198 municipalities, and the representative social worker of each organization responded to one questionnaire. We chose the Kansai region of Japan as the study field because the Kansai region includes several prefectures and municipalities which have the highest proportion of the population on public assistance in the country, such as Osaka and Kyoto. The questionnaire was sent along with a stamped return envelope. In order to ensure easy access to the questionnaire and increase the response rate, we concurrently prepared a web-based questionnaire system as an option (with an index URL and QR code) when distributing the questionnaires by mail. Moreover, we sent a reminder postcard, a week after the deadline to optimize the response rate.

As this study is a part of a larger project to elucidate the financial protection of older people, the cases difficult to support and their management process were asked in detail and were discussed for the improvement by additional in-depth interviews with the survey respondents who actively volunteered to also participate as part of their survey response. These results will be reported in other opportunities due to the limited word counts of this manuscript.

#### Certified Social Workers as Respondents

Regarding the certified social workers in Japan, they are approved under the national qualification of social welfare based on the Law of Certified Social Workers and Care Workers (1987). They are expected to be a key person and to provide consultation, advice, and guidance to those who have difficulties with their daily life due to any types of disability or environmental barrier. More than 260,000 certified social workers were reported to be registered across the country in fiscal year 2021 (Health, Labour and Welfare Statistics Association, 2021a; 2021b; MHLW, 2021b; Iwasaki et al., 2014).

Workplaces could vary (e.g., hospitals, local governments, the community general support centers, or the social welfare councils) according to a social worker’s interests. MedSWs are those who directly support patients and manage the required support for them during hospital admission and after discharge. SWs in local governments or specific care centers support their local residents in daily life issues. Usually, MedSWs and SWs collaborate and consult with each other when needed (Health, Labour and Welfare Statistics Association, 2021a; 2021b; Inaba et al., 2020).

#### Survey Questions

The questionnaire contents were designed to clarify the following elements. First, frequency of the usage of the seventeen protective systems and programs (e.g., “Fringe benefits of a part of Health Insurance Societies/Mutual Aid Associations”, “Reduction, suspension or exemption according to the Article 44 of the National Health Insurance Act”) were asked (See Appendix A). Second, in order to identify the social background of typical clients and specify their major needs, we asked both MedSWs and SWs about (1) the common social background characteristics of the client’s households who requested a consultation, (2) the frequency of consultations regarding the client’s socio-economic background, (3) difficulties in familial backgrounds of the clients, and (4) major difficulties faced by the client themselves, by choosing the top two or three of the multiple-choice items. Third, for MedSW only, we asked about (5) frequent consultations regarding the patient’s diseases and (6) frequent consultations regarding the patient’s therapeutic practices to identify the health care areas in need. Fourth, we asked both MedSWs and SWs about (7) their expected field of policy/program improvement for clients to receive medical care. Lastly, we asked about the demographic characteristics of social workers.

### Statistical Analysis

After descriptive statistics were calculated, the frequency distributions of the respondent’s personal characteristics, such as their gender, social work setting (i.e., hospitals or one of the three other institutions), geographic location/prefecture, and length of professional experience were described in relation to the six most infrequently used financial support programs out of the 17 that were listed in the survey. The “infrequent or no use” of each program was defined as the sum of “not at all” and “1 to 5 people every few years” responses out of the 5-level response (“not at all” to “a few people a week”), and the proportion of respondents was also calculated. Subsequently, we examined the associations between the social worker-level factors such as personal characteristics (i.e., gender, work setting, length of professional experience) and geographic region of their workplace, and the “infrequent or no use” of each protective policy and program as dependent variables using logistic regression analyses. We examined models based on assumptions of two causal relationships to ensure the robustness of findings (Appendix B_ Supplementary Fig. 1a/1b). In order to avoid mutual adjustment fallacy when performing multivariable logistic regression, we identified the variables that required adjustment based on the backdoor criterion using a directed acyclic graph (Pearl et al, 2016). The point estimates of the odds ratios (ORs) from the two regression analyses are shown in Table 4. The point estimates and 95% confidence intervals are reported in the supplementary tables (Appendix B_ Supplementary Table 2a/2b). Data with missing outcome data and no answer data were not included in the analyses, after ensuring that they were less than 10%.

Statistical calculations were conducted using IBM SPSS Statistics for Windows, Version 27.0 (IBM Corp., Armonk, NY). All tests were two-tailed, with a significance level of 0.05.

## Results

A total of 200 MedSWs and 358 SWs responded to the questionnaire (response rate 17.8 and 26.5%, respectively). After excluding those who responded with a completely blank sheet or responded in an unreliable manner, which were ensured by multiple authors, response data from 198 MedSWs and 355 SWs were included in the final analysis. Descriptive statistics of the respondents by MedSWs and SWs are shown in Table 1. The certified social workers and the long-term care support specialists were the main respondents, and more than a half of the respondents were from Osaka and Hyogo.

**Table 1:** Baseline characteristics of the respondent institutions and respondents

	Total		MedSWs		SWs	
	n = 553		n = 198		n = 355	
Institutions n (%)						
Hospitals (including 1 clnic)	191	(34.6)	191	(96.5)	–	–
Community general support center	231	(41.8)	–	–	231	(65.1)
Social welfare council	85	(15.4)	–	–	85	(23.9)
Local government	36	(6.5)	–	–	36	(10.1)
No answer	10	(1.8)	7	(3.5)	3	(0.8)
Region of the institutions n (%)						
Osaka	196	(35.4)	79	(39.9)	117	(33.0)
Hyogo	114	(20.6)	42	(21.2)	72	(20.3)
Kyoto	80	(14.5)	33	(16.7)	47	(13.2)
Shiga	44	(8.0)	9	(4.5)	35	(9.9)
Nara	51	(9.2)	20	(10.1)	31	(8.7)
Wakayama	34	(6.1)	13	(6.6)	21	(5.9)
No answer	34	(6.1)	2	(1.0)	32	(9.0)
Professional qualifications (multiple answers allowed) n (%)						
Certified social worker	398	(72.0)	162	(81.8)	236	(66.5)
Long-term care support specialist	245	(44.3)	73	(36.9)	172	(48.5)
Social welfare officer qualifications	123	(22.2)	40	(20.2)	83	(23.4)
Certified psychiatric social worker	90	(16.3)	35	(17.7)	55	(15.5)
Registered nurse	29	(5.2)	–	–	29	(8.2)
Public health nurse	26	(4.7)	–	–	26	(7.3)
Teacher certification	25	(4.5)	8	(4.0)	17	(4.8)
Other	59	(10.7)	29	(14.6)	30	(8.5)
Years of professional experience median (IQR)	15.0 (8.0–20.0)		14.5 (9.0–20.0)		15.0 (8.0–20.0)	
Age n (%)						
20–29 years	21	(3.8)	8	(4.0)	13	(3.7)
30–39 years	116	(21.0)	45	(22.7)	71	(20.0)
40–49 years	230	(41.6)	96	(48.5)	134	(37.7)
50–59 years	129	(23.3)	39	(19.7)	90	(25.4)
60–69 years	36	(6.5)	7	(3.5)	29	(8.2)
No answer	21	(3.8)	3	(1.5)	18	(5.1)
Gender n (%)						
Female	269	(48.6)	108	(54.5)	161	(45.4)
Male	250	(45.2)	83	(41.9)	167	(47.0)
Non-binary (include no answer)	34	(6.1)	7	(3.5)	27	(7.6)

*MedSW* medical social worker, *SW* social worker in the three institutions such as Community general support centers, Social Welfare Council, and local government

Table 2 shows the responses regarding the clients’ social situation that both MedSWs and SWs experienced in common. The major social problems affecting the older clients, in the order of most to least frequently observed, were, “self and/or cohabiting spouse has dementia”, “poor comprehension of the client/key person”, “social isolation”, “unclear family financial management status”, “self-neglect”, and “cohabiting child is disabled or is severely socially withdrawn (otherwise known as *hikikomori*)” (Kato et al, 2019; Teo, 2010). With regard to the client’s socio-economic background, the two most frequently encountered situations were, “income is at the level eligible for public assistance but has not applied for it” and “delayed payment of long-term care insurance premiums”. The most commonly observed family or household problems were “no primary caregiver to take care of them” and “no family at all”. Concerning the major difficulties with the clients themselves, the three most common cases involved, “poor comprehension of the client”, “refusal to accept various kinds of support”, and “dementia”. In addition, concerning the social worker’s expected field of program improvement for clients to receive medical care, “support for receiving medical care such as accompanied to hospitals by someone in an appropriate way etc.” were ranked to be the top interests.

**Table 2:** Characteristics of the clients’ social situation and a request for program improvement from the respondents

n (%)	MedSWs		SWs	
	n = 198		n = 355	
Major social background of client households*				
Self and/or cohabiting spouse has dementia	185	(93.4)	324	(91.3)
Poor understanding abilities (eg, mental retardation) of the person or someone who cares him/her mainly	182	(91.9)	305	(85.9)
Social isolation (no relatives or acquaintances to rely on even if they exist)	176	(88.9)	306	(86.2)
Unclear how they manage their daily finances and finances	158	(79.8)	297	(83.7)
General lack of motivation in life by the person or someone who cares him/her mainly (i.e. self-neglect)	150	(75.8)	247	(69.6)
Cohabiting child who is disabled/has social withdrawal “*Hikikomori*”	148	(74.7)	285	(80.3)
Being financially abused by cohabiting spouse, child, etc	113	(57.1)	187	(52.7)
Abused (physically, psychologically, sexually, neglected, etc.) by cohabiting spouse or children	103	(52.0)	243	(68.5)
Frequent consultations regarding the client’s socio-behavioral and economic background				
Income is at the level of public assistance but has not applied for it	108	(54.5)	254	(71.5)
Delayed payment of long-term care insurance premiums	102	(51.5)	188	(53.0)
Insurance card taken away due to delayed payment of national health insurance premiums	54	(27.3)	60	(16.9)
Non-regular employment	22	(11.1)	53	(14.9)
No family registration/no insurance	62	(31.3)	33	(9.3)
Job seeking	14	(7.1)	32	(9.0)
Have not applied for insurance (returning from abroad, etc.)	20	(10.1)	23	(6.5)
Other	21	(10.6)	45	(12.7)
Difficulties in familial backgrounds of the clients				
No primary caregiver to take care of them (no one close to them to rely on)	128	(64.6)	247	(69.6)
No family	143	(72.2)	216	(60.8)
Living alone	60	(30.3)	118	(33.2)
Suffering from spousal violence (domestic violence) or abuse	33	(16.7)	64	(18.0)
Homeless	18	(9.1)	14	(3.9)
Other	11	(5.6)	40	(11.3)
Major difficulties of the client themselves				
Poor understanding (mental retardation, etc.)	161	(81.3)	267	(75.2)
Refuses to accept various kinds of support	140	(70.7)	232	(65.4)
Dementia	139	(70.2)	214	(60.3)
Mental disability	57	(28.8)	189	(53.2)
NEET/social withdrawal “*Hikikomori*”	20	(10.1)	80	(22.5)
Foreigner (staying in Japan illegally)	32	(16.2)	12	(27.3)
Hearing, visual, or speech impairment	9	(4.5)	10	(2.8)
Foreigner (with visa status)	6	(3.0)	10	(2.8)
Physical disability	7	(3.5)	3	(0.8)
Other	7	(3.5)	12	(3.4)
Expected field of system/program improvement for clients to receive medical care				
Support for receiving medical care (assistance in a series of processes up to examinations in hospitals, accompaniment, etc.)	146	(73.7)	274	(77.2)
Support for daily life	72	(36.4)	124	(34.9)
Enhanced home-visit medical treatment	60	(30.3)	122	(34.4)
Psychological support	24	(12.1)	62	(17.5)
Support for nursing care	47	(23.7)	30	(8.5)
Support for housing	12	(6.1)	22	(6.2)
Support for employment	6	(3.0)	25	(7.0)
Support for education	9	(4.5)	10	(2.8)
Other	16	(8.1)	21	(5.9)

*NEET* not in education, employment or training

*The proportions of the selected responses of a “a few persons per week”, “a few persons per month” and “a few persons per year”

With regard to the types of healthcare that the older clients need, the three most commonly consulted therapeutic treatments were, “routine treatment of bedsores and other skin diseases at home”, “insulin treatment (chronic with regular dose)”, and “routine injection and supplemental fluid management at home” (Table 3).

**Table 3:** Responses regarding the medical consultations

n (%)	MedSWs	
	n = 198	
Frequent consultations regarding the patient’s diseases*		
Respiratory diseases	171	(86.4)
Cardiovascular diseases	170	(85.9)
End-of-life care (including palliative care)	169	(85.4)
Gastrointestinal diseases	167	(84.3)
Cancer	165	(83.3)
Auto-immune diseases (eg Rheumatoid Arthritis, collagen diseases)	142	(71.7)
Blood diseases	121	(61.1)
Rare diseases	80	(40.4)
Frequent consultations regarding the patient’s therapeutic practices*		
Routine treatment of bedsores and skin diseases at home	165	(83.3)
Insulin (chronic with regular dose)	162	(81.8)
Routine injection and supplemental fluid management at home	147	(74.2)
Blood transfusion	130	(65.7)
Infection control at home (COVID-19, parasites, etc.)	124	(62.6)
Surgery (minor, such as cataract surgery)	116	(58.6)
High-cost drugs (molecular targeted drugs, anticancer drugs, etc.)	110	(55.6)
Hemodialysis (chronic)	103	(52.0)
Surgery (for severe cases using ICU)	99	(50.0)

*The proportions of the selected responses of “a few persons per week”, “a few persons per month” and “a few persons per year” were calculated

Next, we examined the frequencies of the use of major financial aid programs for health care, by personal characteristics of the social worker and by geographic region of their workplace (Appendix B Supplementary Table 1). Further, we explored the relation between each variable and each program by performing a series of univariable and multivariable logistic regression analyses (Table 4, Appendix B Supplementary Table 2a/2b).

**Table 4:** Factors associated with the infrequent or no use of the programs using univariable and multivariable logistic regression

	Mutual Aid System for persons with disabilities		Fringe benefits of a part of Health Insurance Societies/Mutual Aid Associations		Publicly funded free or low-cost medical treatment program		Reduction, suspension or exemption according to the Article 44* of the National Health Insurance Act		Credit for income taxes (deduction for person with disabilities, deduction for medical expenses)		Community Life Support Service (by prefectures/municipalities)	
	OR		OR		OR		OR		OR		OR	
	Univariable	Multivariable	Univariable	Multivariable	Univariable	Multivariable	Univariable	Multivariable	Univariable	Multivariable	Univariable	Multivariable
Gender^a^												
Male	0.909	0.903	1.137	1.127	1.292	1.299	**1.511**	1.436	**1.553**	**1.542**	1.132	1.146
Female	Ref.		Ref.		Ref.		Ref.		Ref.		Ref.	
Non-binary	0.467	0.465	1.220	1.264	0.698	0.714	**0.297**	**0.297**	1.044	1.019	0.472	0.466
Work setting^b^												
MedSW	Ref.		Ref.		Ref.		Ref.		Ref.		Ref.	
SW	**2.309**	**2.556**	**7.959**	**8.160**	1.366	1.267	1.088	1.127	**2.430**	**2.700**	1.327	1.409
Region of the institutions^d^												
Osaka	1.949	1.949	0.764	0.764	0.585	0.585	1.099	1.099	1.343	1.343	1.165	1.165
Hyogo	Ref.		Ref.		Ref.		Ref.		Ref.		Ref.	
Kyoto	0.891	0.891	0.776	0.776	0.604	0.604	1.634	1.634	1.630	1.630	1.207	1.207
Shiga	2.946	2.946	1.513	1.513	1.542	1.542	**3.228**	**3.228**	0.804	0.804	1.050	1.050
Nara	1.744	1.744	1.378	1.378	0.528	0.528	2.460	2.460	1.443	1.443	1.198	1.198
Wakayama	0.749	0.749	1.134	1.134	2.417	2.417	3.197	3.197	1.088	1.088	0.754	0.754
Length of professional experience^c^												
< 10 years	0.933	0.766	1.487	1.438	1.869	1.745	**1.936**	1.663	1.378	1.316	**1.883**	**1.725**
10 to 18 years	Ref.		Ref.		Ref.		Ref.		Ref.		Ref.	
19 years and more	0.657	0.544	1.552	1.503	0.804	0.736	1.385	1.161	1.003	0.924	1.167	1.055

*MedSW* medical social worker, *SW* social worker in the three institutions such as Community general support centers, Social Welfare Council, and local government; *OR* odds ratio, *CI* confidence intervals

*Article 44 of the National Health Insurance Act defines reduction/postponement/exemption based on the judgment of the municipality regarding “people insured for special reasons”

The point estimates of the OR are shown in the table. Statistically significant values are shown in bold

The adjusted variables are ^a^region; ^b^gender and region; and ^c^gender and work setting, in the multivariable regression analyses; ^d^No adjustment required

The result from the regression analyses shows that male social workers, SWs working in one of the three types of social service institutions (i.e., community general support centers, social welfare council, and local government office), and those with less professional experience were associated with the infrequent or no use of the major financial aid programs for healthcare. For example, when focusing on the “Reduction, suspension or exemption (of premium payments) according to the Article 44 of the National Health Insurance Act”, factors related with infrequent or no use of these programs were identified as gender (“male” vs “female”: odds ratio, OR 1.44; 95% CI 0.91–2.28; “non-binary” vs “female”: OR 0.30; 0.14–0.65), and length of professional experience (“less than 10 years” vs “10 to 19 years”: OR 1.66; 0.94–2.95). Odds ratio and 95% CI of each variable are shown in Appendix B_ Supplementary Table 2a/2b.

Similarly, regarding the “Fringe benefits of a part of Health Insurance Societies/Mutual Aid Associations”, work setting (“SWs working in one of the three social service institutions” vs “MedSWs”: OR 8.16; 4.62–14.41) was associated with a significantly higher likelihood of not utilizing these benefits for the client, along with being male and having less than 10 years of professional experience (OR 1.13 and OR 1.44 respectively, compared with each reference). Although these factors were not necessarily significant in association with the use of every program in the survey, these associations were observed across six of the 17 programs. However, almost no patterns were observed in the relationship between geographic region of the workplace and the use of available financial aid programs.

In summary, social workers who identify as male, SWs working in one of the three types of social service institutions, and those with less than 10 years of professional experience were associated with the infrequent or no use of the major financial protection programs available for older persons in need of health care. Meanwhile, in some cases, those identifying as being of “non-binary” gender tended to use some of these programs more often than their female and male counterparts.

## Discussion

This study highlighted the unmet needs for healthcare among older persons, especially financially challenged households which include them, and revealed three crucial points for policy interventions in the future. First, financial problems were often intertwined with social and mental health problems of the client or their household member. Second, many of the current policies and programs of financial protection in Japan are reportedly infrequently used or never used by social workers working at hospitals and local social service institutions to help their clients. Lastly, the personal characteristics of the social workers, namely male gender (compared to female gender), working in social service institutions (compared to working in hospitals) and having less than 10 year of social work experience (compared to 10 years or more) were each associated with the infrequent or no use of several important financial aid programs, whereas social workers of “non-binary” gender were observed to apply some of these programs more frequently.

### Who May Be Left Behind?

In this study, social and mental health problems of the clients themselves or their household members were revealed to be the biggest challenges that underlie their financial problems. For example, according to the surveyed social workers, clients and/or their household members exhibited refusal of any kind of support, physical and mental disabilities, poor comprehension, severe social isolation, and unemployment, making it difficult for medical and social work professionals to communicate with them and provide them with appropriate support.

Difficulties in the household environment included not having a key person or relatives to provide care for the client, poor social capital, and living alone/isolation. Additionally, in terms of socioeconomic background, households at risk of impoverishment who did not apply for public assistance or who delayed payments of long-term care insurance premiums were the most common situations observed in which a client may have difficulty paying for health care and may go without needed care (Table 2). This subset of the population could easily be left behind because their voices are too weak and they are hardly noticed by social workers and neighbors. As part of a solution, it is reported that it is desirable to first be aware of their existence and create a system or ongoing activities to support them as a community (Iwasaki et al., 2014; Okuda et al., 2021). This population highlighted in this study is consistent with the previous studies (Iwasaki et al., 2014; Okuda et al., 2021) and we successfully visualized them with numbers.

Our findings further revealed the unmet healthcare needs of financially struggling households in detail, and may add some value in addition to the international reports on financial protection in healthcare which use the microdata from household budget surveys (WHO, 2019a; WHO, 2018), global monitoring report on financial protection in health (WHO and IBRD/The World Bank, 2021) and WHO Health Equity Policy Tool (WHO, 2019b), especially regarding the measures of “2.1 Health and health services” and “2.2 Health and income security and social protection” (WHO, 2019b).

To note, expensive medical care, such as molecular targeted drugs and anticancer drugs, were not found to be among the most frequently reported reasons for needing financial aid for healthcare, as the high-cost medical expense benefit, which refunds patients for healthcare expenditures exceeding a set monthly threshold, is reportedly well utilized and disseminated at a national level (Table 3).Instead, therapeutic treatment for chronic conditions, such as bedsores and other skin ailments as well as the daily use of insulin, were the more common treatments for which clients required financial aid. For example, it could be an economic burden for some patients to pay for medical materials such as the large volume of gauzes used for routine treatment of bedsores and skin diseases at home. Needles for insulin could also be a burden for other patients. Therefore, these would be potential areas for financial protection.

When focusing on social worker’s expected field of program improvement for clients to receive medical care, “support for receiving medical care such as accompanied to hospitals by someone in an appropriate way etc.” was ranked to be the top interest (Table 2). Given the growing population of households facing complex and diversified issues such as “cohabiting child who is disabled/has social withdrawal *hikikomori*”, “unmarried children in their 50 s who are unemployed live with their parents in their 80 s (often referred to as the ‘8050 problem’)”, and “parents of disabled persons who are aging and need nursing care”, the Japanese government is encouraging “cooperation among different organizations in the municipal level to build a comprehensive consultation system for people who need assistance and advice” (MHLW, 2018). The results of our study could be aligned to this context so that (1) clients who could not express their needs for medical care would be the future target population for policy intervention, and (2) these patients could receive appropriate care not only of aid for hospital discharge but also of assistance in the process of going to hospitals.

### Policies and Programs that Tend to be Underused and Their Associated Factors

Although it is natural to see some variation in the different aspects of social work support such as MedSWs and SWs, the most infrequently applied policies and programs in common included those of the direct aid for medical expenses (e.g., “Fringe benefits of a part of Health Insurance Societies/Mutual Aid Associations”, “Publicly funded free or low-cost medical treatment program”, “Reduction, suspension or exemption according to the Article 44 of the National Health Insurance Act”, and “Credit for income taxes”), those for disabilities (e.g., “Mutual Aid System for persons with disabilities”), and those to aid daily decision of financial management or daily living issues in need (e.g., “Community Life Support Service by prefectures or municipalities”) (Appendix B_ Supplementary Table 1). These systems could be utilized more but seemed complicated and inconvenient to use. Previous reports and studies have denoted the system-level factors of complex, fragmented programs including the difficult application process as one reason for their underutilization (Komatsu, 2018; Nishigaki, 2011; Sato, 2014). In short, the active use of programs that mainly depend on the preparedness of the local governments, hospitals, or social work professionals in charge, seemed to vary significantly across regions and institutions. Flexible systems such as cross-sectoral support for social and mental hardship of the clients and their households are necessary for the financial protection policies to be universally effective.

Regarding “Publicly funded free or low-cost medical treatment program”, Japanese government statistics reported regional variations in the number of hospitals and clinics that adopted this program (MHLW, 2018). Although a relatively high number of institutions adopted this program in accordance with the highest needs of public assistance in Kansai region, perceptions of this program seem to have remained low among MedSWs and SWs (Appendix B_ Supplementary Table 1). This could be due to a hospital-level factor by which poor financial incentives are given to general hospitals such that adoption of the program tends to be biased to specific hospital groups which have a high aspiration to protect the move vulnerable people. (Yoshinaga, 2019; Japanese Government Statistics, 2017).

This study further revealed the personal factors of the social workers related with the infrequent use of many of these financial aid programs. We identified that being male, being SWs (who work at community general support centers, social welfare councils and local governments), and having less than 10 years of professional experience were associated with the infrequent use of a number of the programs asked about in the survey. Meanwhile, social workers of “non-binary” gender, though comprising only 6% of the respondents, tended to use some of these programs more often than their female or male counterpart (Table 4). A possible explanation for this could be that “non-binary” gender may have a higher level of empathy with minority populations, which may lead to them being more proactive in their search for and use of applicable programs for their clients. The finding of the “non-binary” gender may be overlooked in the international context of gender and social work such as the debate focused on female-dominated in number but smaller number of men holding more institutional power (Hicks, 2015; Jones et al, 2019). Moreover, few studies are available which have examined the association between the individual backgrounds of social workers and quality of service provision, except in the context of work-related stress and career experiences (Japanese Government Statistics, 2017; Matsuda, 2016). Therefore, further studies are needed to clarify the mechanism by which the personal factors of social workers affect their utilization of specific financial aid programs and their quality of service more broadly.

### Implications for Policy Interventions in the Practice of Social Work

As shown from our study, financial protection policies and programs are not necessarily utilized frequently by the two types of social workers. Therefore, basic protective knowledge, notices, and related updated information need to be conveyed to both social workers and clients in an easily accessible manner. Policy makers should be aware of that simple bureaucratic notices about the policies and programs through paper copies or online are not enough.

In addition, our novel findings may contribute to the next generation of the frameworks of health equity, that focus on provider-level factors, such as the characteristics of social workers who directly support clients (WHO, 2019b; Park, 2020; Penhale et al., 2015; Kwong, 2018). Social workers experience multiple contradictions in the process of their daily service delivery based on their diverse characteristics such as age, gender, race, and social position (Baines, 2017; Hulko et al., 2019). When considering the implementation of justice, equity, diversity, and inclusion in practice, every social worker should be trained in a range of justice-oriented approaches, including anti-oppressive practice, which is one of the main social justice-oriented social work theory today (Baines, 2017). Particularly, in the context of caring for older adults in an era of austerity, rethinking care providers’ conventional stereotypes of older adults such as passive clients of care and over-reliance on unpaid family care, will be a difficult but crucial process of change—from the frontline to agency policy (Baines, 2017). As such, social work could be cultivated from the micro (individual) to the meso (community) to the macro (global) level of practice (Baines, 2017). This training approach, which includes anti-oppressive gerontology, has the potential to produce highly skilled practitioners with a place for older clients, communities, and broader social justice struggles (Baines, 2017; Hulko et al., 2019). In this context, our findings are consistent with the need for recent graduates and new frontline social workers to receive ongoing training that takes into account their own backgrounds, experiences, and values, based on a sound system of lifelong education, which includes anti-oppressive gerontological approaches, in order to achieve true client-centered care.

### Limitations

Several potential limitations should be noted. First, relatively low response rate, especially by SWs working in local government may have biased the results, though among the institutions included in the survey, local government offices are generally not the main point of contact for clients who need financial aid for medical care in Japan. Second, potential bias could arise from the self-reported data, when it comes to the client detailed issue, but our focus was mainly to detect the objective situation and the program use which would not change the results. Third, the region was limited to Kansai, which may limit the generalizability of the findings to the rest of Japan or to other countries. However, the Kansai region includes several prefectures and municipalities which have the highest proportion of public assistance in the country. By focusing on the Kansai region, we ensured that there would be enough experience and knowledge about the population facing challenges in paying for healthcare and that the study results would be relevant to the community (MHLW, 2021a; Japanese Government Statistics, 2017). Lastly, discriminatory aspects of some welfare policies which may hinder the older adults’ access to services are not fully identified in this study. This could be further investigated in the future research.

Despite these limitations, we believe that the study contributes new knowledge to the implementation gaps in the financial protection policy and system of older persons in health care. Because individual social workers are the direct implementers of policies and programs, and are likely to be the bridge between individuals/households in need and sufficient care, a detailed clarification of the policy usage in a practical setting may contribute to the next generation of conceptual frameworks to effectively implement important policies.

## Conclusions

Many financial challenges were revealed to be intertwined with the social and mental health issues of clients themselves and/or their households. Moreover, male, social workers at local offices/agencies, and less than 10 years’ professional experience associated with infrequent or no use of key protective programs. To close the gap between policy and practice, policies should focus on clients’ daily living needs, and new frontline social workers should receive lifelong training that incorporates their own backgrounds, experiences, and values, including the use of anti-oppressive gerontological approaches.

### Supplementary Information

Below is the link to the electronic supplementary material.

Supplementary file1 (DOCX 41 KB)

Supplementary file2 (DOCX 82 KB)

## Data Availability

The data that support the findings of this study are available from the corresponding author upon request.

## Abbreviations

LTCI: Long term care insurance
MedSW: Medical social worker
NHI: National health insurance
SW: Social worker
UHC: Universal health coverage
