# Supplementary material for: Hidden Populations for Healthcare Financial Protection in the Super-Aging Society: Closing the Gap Between Policy and Practice
Source: Clin Soc Work J. 2024 Jan 16;52(3):310–21. doi: 10.1007/s10615-023-00914-x (PMC11344706; doi:10.1007/s10615-023-00914-x)
Supplement: Supplementary file 1 — Supplementary file1 (DOCX 41 KB) [file 10615_2023_914_MOESM1_ESM.docx]

Questionnaire 1. Contents of the questionnaire survey for MedSW

Q. To what extent do you use the following systems and programs daily in your hospital? Please select **one for each**. Please enter the number of people you provided support to, and not the number of consultations. Also, if there is a great need to use the system but you can't use it, please put a check mark (☑) in the right column as well.

|  | Not at all | 1～5 persons per a few years | A few persons per year | A few persons per month | A few persons per week | **There is a great need but is not always available.** |
| --- | --- | --- | --- | --- | --- | --- |
| 1.Credit for income taxes（deduction for person with disabilities、deduction for medical expenses） | □ | □ | □ | □ | □ | □ |
| 2. Medical subsidy for rare disease, or for specific disease of infants and children | □ | □ | □ | □ | □ | □ |
| 3. High-cost medical expense benefit（Eligibility Certificate for Ceiling-Amount Application） | □ | □ | □ | □ | □ | □ |
| 4.Fringe benefits of a part of Health Insurance Societies / Mutual Aid Associations | □ | □ | □ | □ | □ | □ |
| 5. Publicly funded free or low-cost medical treatment program | □ | □ | □ | □ | □ | □ |
| 6. Reduction, suspension or exemption according to the Article 44* of the National Health Insurance Act | □ | □ | □ | □ | □ | □ |
| 7.Application for welfare book（people with mental disorders, or people with physical disabilities） | □ | □ | □ | □ | □ | □ |
| 8.Public Assistance System | □ | □ | □ | □ | □ | □ |
| 9.Long-term Care Insurance System (application or change request） | □ | □ | □ | □ | □ | □ |
| 10. Pension (Disability Basic Pension, Disability Employees’ Pension, Disability Mutual Aid Pension) | □ | □ | □ | □ | □ | □ |
| 11. Medical System for Services and Supports for Persons with Disabilities | □ | □ | □ | □ | □ | □ |
| 12. Self-reliance Support for Needy Persons | □ | □ | □ | □ | □ | □ |
| 13. Support Program for Self-Reliance in Daily Life | □ | □ | □ | □ | □ | □ |
| 14. Community Life Support Service (by prefectures/ municipalities) | □ | □ | □ | □ | □ | □ |
| 15. Mutual Aid System for persons with mental or physical disabilities | □ | □ | □ | □ | □ | □ |
| 16.Adult Guardianship system(voluntary／legal) | □ | □ | □ | □ | □ | □ |
| 17. Supporting programs related to COVID-19 | □ | □ | □ | □ | □ | □ |

* Article 44* of the National Health Insurance Act allows for reductions, suspensions, and exemptions at the discretion of the municipality for insured persons who are under special circumstances.

Q To what extent does your local government (department)/institution receive consultations on the following **social background** of the user's **household**? Please indicate the number of people you have provided support to, not the number of consultations.

However, if a person has multiple issues, please count each item separately. (For example, if one person has three issues, count one person for each of the three items.)

|  | Not at all | 1～5 persons per a few years | A few persons per year | A few persons per month | A few persons per week |
| --- | --- | --- | --- | --- | --- |
| 1. Self and/or cohabiting spouse has dementia | □ | □ | □ | □ | □ |
| 2. Cohabiting child who is disabled/has social withdrawal ”Hikikomori” | □ | □ | □ | □ | □ |
| 3. Being financially abused by cohabiting spouse, child, etc. | □ | □ | □ | □ | □ |
| 4. Abused (physically, psychologically, sexually, neglected, etc.) by cohabiting spouse or children | □ | □ | □ | □ | □ |
| 5. Intellectual disabilities (eg, mental retardation) of the person or someone who cares him/her mainly | □ | □ | □ | □ | □ |
| 6. Unclear how they manage their daily finances | □ | □ | □ | □ | □ |
| 7. General lack of motivation in life by the person or someone who cares him/her mainly（i.e. self-neglect） | □ | □ | □ | □ | □ |
| 8. Social isolation（no relatives or acquaintances to rely on even if they exist） | □ | □ | □ | □ | □ |

Q. What in particular about the **user's familial background** makes it difficult, including financial difficulties, for them to use the existing social welfare system or public programmes? Please select **two** from those you think are most common.

□1. Homeless □2. Living alone □3. No family

□4. No primary caregiver to take care of them (no one close to them to rely on)

□5. Suffering from spousal violence (domestic violence) or abuse

□6. Other [ ]

Q. What in particular about the **user themselves** makes it difficult, including financial difficulties, for them to use the existing social welfare system or public programmes? Please select **three** from those you think are most common.

□1. NEET：not in education, employment or training/ Social withdrawal ”Hikikomori”

□2. Intellectual disabilities (mental retardation, etc.)

□3. Hearing, visual, or speech impairment □4. Dementia

□5. Mental disability □6. Physical disability

□7. Foreigner (with visa status)　□8. Foreigner (staying in Japan illegally)

□9. Refuses to accept various kinds of support

□10. Other [ ]

Q. What in particular about the **user's social conditions** that makes it difficult, including financial difficulties, for them to use the existing social welfare system or public programmes? Please select **two** from those you think are most common.

□1. Non-regular employment □2. Job seeking

□3. Have not applied for insurance (returning from abroad, etc.)

□4. No family registration/no insurance

□5. Insurance card taken away due to delayed payment of National Health Insurance premiums

□6. Del payment of long-term care insurance premiums

□7. Income is at the level of public assistance but has not applied for it

□8. Other [ ]

Q. How often in the whole hospital do you receive consultation from patients with the following diseases (1-8)?

|  | None | 1～5 persons per a few years | A few persons per year | A few persons per month | A few persons per week |
| --- | --- | --- | --- | --- | --- |
| 1. Blood diseases | □ | □ | □ | □ | □ |
| 2. Cancer | □ | □ | □ | □ | □ |
| 3. Auto-immune diseases (eg Rheumatoid Arthritis, collagen diseases) | □ | □ | □ | □ | □ |
| 4. Gastrointestinal diseases | □ | □ | □ | □ | □ |
| 5. Cardiovascular diseases (heart and blood vessel diseases) | □ | □ | □ | □ | □ |
| 6. Respiratory diseases | □ | □ | □ | □ | □ |
| 7. Rare diseases | □ | □ | □ | □ | □ |
| 8. End-of-life care (including palliative care) | □ | □ | □ | □ | □ |

Q. How often in the whole hospital do you receive consultation from patients receive the following therapeutic practices (1-9)?

|  | None | 1～5 persons per a few years | A few persons per year | A few persons per month | A few persons per week |
| --- | --- | --- | --- | --- | --- |
| 1. High-cost drugs (molecular targeted drugs, anticancer drugs, etc） | □ | □ | □ | □ | □ |
| 2. Blood transfusion | □ | □ | □ | □ | □ |
| 3. Surgery（for severe cases using ICU） | □ | □ | □ | □ | □ |
| 4. Surgery（minor, such as cataract surgery） | □ | □ | □ | □ | □ |
| 5. Insulin（chronic with regular dose） | □ | □ | □ | □ | □ |
| 6. Hemodialysis (chronic) | □ | □ | □ | □ | □ |
| 7. Routine injection and supplemental fluid management at home | □ | □ | □ | □ | □ |
| 8. Routine treatment of bedsores and skin diseases at home | □ | □ | □ | □ | □ |
| 9.Infection control at home (COVID-19, parasites, etc） | □ | □ | □ | □ | □ |

Q. What kinds of **support and system**s do you think could be strengthened to make it easier for users to receive medical care? Please select **two** from those you think are most common.

　　□1. Support for receiving medical care constantly (assistance in a series of processes up to medical examination, accompaniment, etc.)

□2. Enhanced home-visit medical treatment

□3. Support for daily life　□4. Support for nursing care　□5. Support for housing

□6. Support for employment　□7. Support for education　□8.Psychological support

□9.Other 　[ 　　　　　　　　　　　　　　　　　　　　　　　　　 ]

Q. Lastly, we would like to ask you about yourself.

(S1) Gender: □1. Male □2. Female □3. Other □4. I do not want to answer.

(S2) Age: □1. 20-29 □2. 30-39 □3. 40-49 □4. 50-59 □5. 60-69 □6. 70 or older

(S3) Please check one if you hold a license/discipline/degree. *(Multiple answers allowed)*

If you have any professional qualifications, please select them. (Multiple answers are acceptable)

□ 1.Certified Social Worker □2. Certified Psychiatric Social Worker □3. Teacher certification

□4. Long-term Care Support Specialist □ 5. Social Welfare Officer Qualifications

□6. Other [ ]

(S4) How many years of experience do you have in the social welfare field? [ ] years

Questionnaire 2. Contents of the questionnaire survey for SW working at local government, Social Welfare Council, and community general support center

Q. The facility where you work：

（A0）Please select your workplace.

□1. local government □2. Social Welfare Council □3. Community general support center

（A1）Location of your workplace：

(A1-1) Prefecture [ ]

(A1-2) □ 1.Ordinance-designated City □2. Core city □3. Other [ ]

Q. To what extent do you use the following systems and programs daily in your local government (department) / organization?

Please select **one for each**. Please enter the number of people you provided support to, and not the number of consultations. Also, if there is a great need to use the system but you can't use it, please put a check mark (☑) in the right column as well.

|  | Not at all | 1～5 persons per a few years | A few persons per year | A few persons per month | A few persons per week | **There is a great need but not always available.** |
| --- | --- | --- | --- | --- | --- | --- |
| 1.Credit for income taxes（deduction for person with disabilities、deduction for medical expenses） | □ | □ | □ | □ | □ | □ |
| 2. Medical subsidy for rare disease, or for specific disease of infants and children | □ | □ | □ | □ | □ | □ |
| 3. High-cost medical expense benefit（Eligibility Certificate for Ceiling-Amount Application） | □ | □ | □ | □ | □ | □ |
| 4.Fringe benefits of a part of Health Insurance Societies / Mutual Aid Associations | □ | □ | □ | □ | □ | □ |
| 5. Publicly funded free or low-cost medical treatment program | □ | □ | □ | □ | □ | □ |
| 6. Reduction, suspension or exemption according to the Article 44* of the National Health Insurance Act | □ | □ | □ | □ | □ | □ |
| 7.Application for welfare book（people with mental disorders, or people with physical disabilities） | □ | □ | □ | □ | □ | □ |
| 8.Public Assistance System | □ | □ | □ | □ | □ | □ |
| 9.Long-term Care Insurance System (application or change request） | □ | □ | □ | □ | □ | □ |
| 10. Pension (Disability Basic Pension, Disability Employees’ Pension, Disability Mutual Aid Pension) | □ | □ | □ | □ | □ | □ |
| 11. Medical System for Services and Supports for Persons with Disabilities | □ | □ | □ | □ | □ | □ |
| 12. Self-reliance Support for Needy Persons | □ | □ | □ | □ | □ | □ |
| 13. Support Program for Self-Reliance in Daily Life | □ | □ | □ | □ | □ | □ |
| 14. Community Life Support Service (by prefectures/ municipalities) | □ | □ | □ | □ | □ | □ |
| 15. Mutual Aid System for persons with mental or physical disabilities | □ | □ | □ | □ | □ | □ |
| 16.Adult Guardianship system (legal) | □ | □ | □ | □ | □ | □ |
| 17. Adult Guardianship system (voluntary) | □ | □ | □ | □ | □ | □ |
| 18. Supporting programs related to COVID-19 | □ | □ | □ | □ | □ | □ |

* Article 44* of the National Health Insurance Act allows for reductions, suspensions, and exemptions at the discretion of the municipality for insured persons who are under special circumstances.

Q. To what extent does your local government (department)/institution receive consultations on the following **social background** of the user's **household**? Please indicate the number of people you have provided support to, not the number of consultations.

However, if a person has multiple issues, please count each item separately. (For example, if one person has three issues, count one person for each of the three items.)

|  | Not at all | 1～5 persons per a few years | A few persons per year | A few persons per month | A few persons per week |
| --- | --- | --- | --- | --- | --- |
| 1. Self and/or cohabiting spouse has dementia | □ | □ | □ | □ | □ |
| 2. Cohabiting child who is disabled/has social withdrawal ”Hikikomori” | □ | □ | □ | □ | □ |
| 3. Being financially abused by cohabiting spouse, child, etc. | □ | □ | □ | □ | □ |
| 4. Abused (physically, psychologically, sexually, neglected, etc.) by cohabiting spouse or children | □ | □ | □ | □ | □ |
| 5. Intellectual disabilities (eg, mental retardation) of the person or someone who cares him/her mainly | □ | □ | □ | □ | □ |
| 6. Unclear how they manage their daily finances | □ | □ | □ | □ | □ |
| 7. General lack of motivation in life by the person or someone who cares him/her mainly（i.e. self-neglect） | □ | □ | □ | □ | □ |
| 8.Social isolation（no relatives or acquaintances to rely on even if they exist） | □ | □ | □ | □ | □ |

Q. What in particular about the **user's familial background** makes it difficult, including financial difficulties, for them to use the existing social welfare system or public programmes? Please select **two** from those you think are most common.

□1. Homeless □2. Living alone □3. No family

□4. No primary caregiver to take care of them (no one close to them to rely on)

□5. Suffering from spousal violence (domestic violence) or abuse

□6. Other [ ]

Q. What in particular about the **user themselves** makes it difficult, including financial difficulties, for them to use the existing social welfare system or public programmes? Please select **three** from those you think are most common.

□1. NEET：not in education, employment or training/ Social withdrawal ”Hikikomori”

□2. Intellectual disabilities (mental retardation, etc.)

□3. Hearing, visual, or speech impairment □4. Dementia

□5. Mental disability □6. Physical disability

□7. Foreigner (with visa status)　□8. Foreigner (staying in Japan illegally)

□9. Refuses to accept various kinds of support

□10. Other [ ]

Q. What in particular about the **user's social conditions** that makes it difficult, including financial difficulties, for them to use the existing social welfare system or public programmes? Please select **two** from those you think are most common.

□1. Non-regular employment □2. Job seeking

□3. Have not applied for insurance (returning from abroad, etc.)

□4. No family registration/no insurance

□5. Insurance card taken away due to delayed payment of National Health Insurance premiums

□6. Delayed payment of long-term care insurance premiums

□7. Income is at the level of public assistance but has not applied for it

□8. Other [ ]

Q. What kinds of **support and system**s do you think could be strengthened to make it easier for users to receive medical care? Please select **two** from those you think are most common.

　　□1. Support for receiving medical care constantly (assistance in a series of processes up to medical examination, accompaniment, etc.)

□2. Enhanced home-visit medical treatment

□3. Support for daily life　□4. Support for nursing care　□5. Support for housing

□6. Support for employment　□7. Support for education　□8.Psychological support

□9.Other 　[ 　　　　　　　　　　　　　　　　　　　　　　　　　 ]

Q. Lastly, we would like to ask you about yourself.

(S1) Gender: □1. Male □2. Female □3. Other □4. I do not want to answer.

(S2) Age: □1. 20-29 □2. 30-39 □3. 40-49 □4. 50-59 □5. 60-69 □6. 70 or older

(S3) If you have any professional qualifications, please select them. (Multiple answers are acceptable)

□ 1.Certified Social Worker □2. Certified Psychiatric Social Worker □3. Teacher certification

□4. Long-term Care Support Specialist □ 5. Social Welfare Officer Qualifications

□6. Public Health Nurse 　□7. Nurse

□8. Other [ ]

(S4) How many years of experience do you have in the social welfare field? [ ] years
