# Supplementary material for: Hidden Populations for Healthcare Financial Protection in the Super-Aging Society: Closing the Gap Between Policy and Practice
Source: Clin Soc Work J. 2024 Jan 16;52(3):310–21. doi: 10.1007/s10615-023-00914-x (PMC11344706; doi:10.1007/s10615-023-00914-x)

Supplementary Table 1. Frequencies of the infrequent or no use of major financial aid programs for health care, by personal characteristics of the social worker and by geographic region of their workplace

| n (%) | Total | Mutual Aid System for persons with disabilities | Fringe benefits of a part of Health Insurance Societies / Mutual Aid Associations | Publicly funded free or low-cost medical treatment program | Reduction, suspension or exemption according to the Article 44* of the National Health Insurance Act | Credit for income taxes（deduction for person with disabilities, deduction for medical expenses） | Community Life Support Service (by prefectures/ municipalities) |
| --- | --- | --- | --- | --- | --- | --- | --- |
| *Gender* |  |  |  |  |  |  |  |
| Male | 244 | 219 (89.8) | 198 (81.1) | 197 (80.7) | 193 (79.1) | 175 (71.7) | 161 (66.0) |
| Female | 275 | 241 (87.6) | 210 (76.4) | 206 (74.9) | 195 (70.9) | 171 (62.2) | 166 (60.4) |
| Non-binary | 34 | 26 (76.5) | 25 (73.5) | 23 (67.6) | 15 (44.1) | 21 (61.8) | 14 (41.2) |
| *Work setting* |  |  |  |  |  |  |  |
| MedSW | 198 | 169 (85.4) | 123 (62.1) | 148 (74.7) | 141 (71.2) | 108 (54.5) | 117 (59.1) |
| SW | 355 | 317 (89.3) | 310 (87.3) | 278 (78.3) | 262 (73.8) | 259 (73.0) | 224 (63.1) |
| *Region of the institutions* | | | |  |  |  |  |
| Osaka | 196 | 176 (89.8) | 150 (76.5) | 144 (73.5) | 137 (69.9) | 133 (67.9) | 122 (62.2) |
| Hyogo | 114 | 99 (86.8) | 88 (77.2) | 96 (84.2) | 83 (72.8) | 73 (64.0) | 69 (60.5) |
| Kyoto | 80 | 69 (86.3) | 60 (75.0) | 58 (72.5) | 62 (77.5) | 56 (70.0) | 51 (63.7) |
| Shiga | 44 | 38 (86.4) | 36 (81.8) | 37 (84.1) | 35 (79.5) | 26 (59.1) | 27 (61.4) |
| Nara | 51 | 45 (88.2) | 41 (80.4) | 38 (74.5) | 40 (78.4) | 35 (68.6) | 33 (64.7) |
| Wakayama | 34 | 29 (85.3) | 27 (79.4) | 29 (85.3) | 26 (76.5) | 22 (64.7) | 18 (52.9) |
| *Length of professional experience* | | | |  |  |  |  |
| <10 years | 145 | 130 (89.7) | 116 (80.0) | 119 (82.1) | 114 (78.6) | 102 (70.3) | 101 (69.7) |
| 10 to 18 years | 186 | 167 (89.8) | 139 (74.7) | 147 (79.0) | 137 (73.7) | 121 (65.1) | 114 (61.3) |
| 19 years and more | 182 | 157 (86.3) | 148 (81.3) | 133 (73.1) | 132 (72.5) | 117 (64.3) | 110 (60.4) |

MedSW: medical social worker; SW: social worker in the three institutions such as Community general support centers, Social Welfare Council, and local government.

*Article 44 of the National Health Insurance Act defines reduction/postponement/exemption based on the judgment of the municipality regarding

“people insured for special reasons.”

|  | Mutual Aid System for persons with disabilities | | Fringe benefits of a part of Health Insurance Societies / Mutual Aid Associations | | Publicly funded free or low-cost medical treatment program | | Reduction, suspension or exemption according to the Article 44* of the National Health Insurance Act | | Credit for income taxes（deduction for person with disabilities, deduction for medical expenses） | | Community Life Support Service (by prefectures/ municipalities) | |
| --- | --- | --- | --- | --- | --- | --- | --- | --- | --- | --- | --- | --- |
|  | *OR* | *95%CI* | *OR* | *95%CI* | *OR* | *95%CI* | *OR* | *95%CI* | *OR* | *95%CI* | *OR* | *95%CI* |
| *Gender^a^* |  |  |  |  |  |  |  |  |  |  |  |  |
| Male | 0.903 | 0.404-2.019 | 1.127 | 0.683-1.860 | 1.299 | 0.808-2.090 | 1.436 | 0.905-2.279 | **1.542** | **1.030-2.307** | 1.146 | 0.777-1.689 |
| Female | ref | | ref | | ref | | ref | | ref | | ref | |
| Non-binary | 0.465 | 0.123-1.758 | 1.264 | 0.416-3.837 | 0.714 | 0.299-1.750 | **0.297** | **0.135-0.652** | 1.019 | 0.458-2.270 | 0.466 | 0.215-1.013 |
| *Work setting^b^* |  |  |  |  |  |  |  |  |  |  |  |  |
| MedSW | ref | | ref | | ref | | ref | | ref | | ref | |
| SW | **2.556** | **1.169-5.591** | **8.160** | **4.620-14.411** | 1.267 | 0.794-2.023 | 1.127 | 0.713-1.781 | **2.700** | **1.797-4.056** | 1.409 | 0.955-2.079 |
| *Region of the institutions^d^* |  |  |  |  |  |  |  |  |  |  |  |  |
| Osaka | 1.949 | 0.723-5.257 | 0.764 | 0.417-1.399 | 0.585 | 0.325-1.053 | 1.099 | 0.666-1.811 | 1.343 | 0.832-2.166 | 1.165 | 0.729-1.863 |
| Hyogo | ref | | ref | | ref | | ref | | ref | | ref | |
| Kyoto | 0.891 | 0.311-2.556 | 0.776 | 0.361-1.655 | 0.604 | 0.292-1.252 | 1.634 | 0.820-3.254 | 1.630 | 0.855-3.108 | 1.207 | 0.661-2.206 |
| Shiga | 2.946 | 0.365-23.750 | 1.513 | 0.486-4.713 | 1.542 | 0.496-4.796 | **3.228** | **1.075-9.691** | 0.804 | 0.394-1.642 | 1.050 | 0.504-2.186 |
| Nara | 1.744 | 0.368-8.264 | 1.378 | 0.486-3.908 | 0.528 | 0.236-1.178 | 2.460 | 0.965-6.266 | 1.443 | 0.686-3.033 | 1.198 | 0.593-2.418 |
| Wakayama | 0.749 | 0.194-2.895 | 1.134 | 0.359-3.590 | 2.417 | 0.534-10.929 | 3.197 | 0.915-11.187 | 1.088 | 0.477-2.484 | 0.754 | 0.341-1.667 |
| *Length of professional experience^c^* | |  |  |  |  |  |  |  |  |  |  |  |
| <10 years | 0.766 | 0.272-2.159 | 1.438 | 0.745-2.778 | 1.745 | 0.912-3.337 | 1.663 | 0.938-2.950 | 1.316 | 0.792-2.187 | **1.725** | **1.055-2.821** |
| 10 to 18 years | ref | | ref | | ref | | ref | | ref | | ref | |
| 19 years and more | 0.544 | 0.218-1.357 | 1.503 | 0.811-2.786 | 0.736 | 0.442-1.226 | 1.161 | 0.702-1.922 | 0.924 | 0.584-1.481 | 1.055 | 0.682-1.631 |

Supplementary Table 2a. Factors associated with the infrequent or no use of the programs using multivariable logistic regression

MedSW: medical social worker; SW: social worker in the three institutions such as Community general support centers, Social Welfare Council, and local government; OR: odds ratio; CI: confidence intervals.

*Article 44 of the National Health Insurance Act defines reduction/postponement/exemption based on the judgment of the municipality regarding “people insured for special reasons.”

Statistically significant values are shown in bold.

The adjusted variables are ^a^ region; ^b^ gender and region; and ^c^ gender and work setting.  ^d^ No adjustment required.

|  | Mutual Aid System for persons with disabilities | | Fringe benefits of a part of Health Insurance Societies / Mutual Aid Associations | | Publicly funded free or low-cost medical treatment program | | Reduction, suspension or exemption according to the Article 44* of the National Health Insurance Act | | Credit for income taxes（deduction for person with disabilities, deduction for medical expenses） | | Community Life Support Service (by prefectures/ municipalities) | |
| --- | --- | --- | --- | --- | --- | --- | --- | --- | --- | --- | --- | --- |
|  | *OR* | *95%CI* | *OR* | *95%CI* | *OR* | *95%CI* | *OR* | *95%CI* | *OR* | *95%CI* | *OR* | *95%CI* |
| *Gender* |  |  |  |  |  |  |  |  |  |  |  |  |
| Male | 0.909 | 0.412-2.003 | 1.137 | 0.694-1.864 | 1.292 | 0.810-2.063 | 1.511 | 0.959-2.381 | **1.553** | **1.044-2.312** | 1.132 | 0.772-1.658 |
| Female | ref | | ref | | ref | | ref | | ref | | ref | |
| Non-binary | 0.467 | 0.125-1.748 | 1.220 | 0.403-3.692 | 0.698 | 0.295-1.652 | **0.297** | **0.137-0.645** | 1.044 | 0.471-2.316 | 0.472 | 0.218-1.024 |
| *Work setting* |  |  |  |  |  |  |  |  |  |  |  |  |
| MedSW | ref | | ref | | ref | | ref | | ref | | ref | |
| SW | **2.309** | **1.065-4.913** | **7.959** | **4.562-13.866** | 1.366 | 0.868-2.150 | 1.088 | 0.703-1.684 | **2.430** | **1.645-3.590** | 1.327 | 0.909-1.936 |
| *Region of the institutions* |  |  |  |  |  |  |  |  |  |  |  |  |
| Osaka | 1.949 | 0.723-5.257 | 0.764 | 0.417-1.399 | 0.585 | 0.325-1.053 | 1.099 | 0.666-1.811 | 1.343 | 0.832-2.166 | 1.165 | 0.729-1.863 |
| Hyogo | ref | | ref | | ref | | ref | | ref | | ref | |
| Kyoto | 0.891 | 0.311-2.556 | 0.776 | 0.361-1.655 | 0.604 | 0.292-1.252 | 1.634 | 0.820-3.254 | 1.630 | 0.855-3.108 | 1.207 | 0.661-2.206 |
| Shiga | 2.946 | 0.365-23.750 | 1.513 | 0.486-4.713 | 1.542 | 0.496-4.796 | **3.228** | **1.075-9.691** | 0.804 | 0.394-1.642 | 1.050 | 0.504-2.186 |
| Nara | 1.744 | 0.368-8.264 | 1.378 | 0.486-3.908 | 0.528 | 0.236-1.178 | 2.460 | 0.965-6.266 | 1.443 | 0.686-3.033 | 1.198 | 0.593-2.418 |
| Wakayama | 0.749 | 0.194-2.895 | 1.134 | 0.359-3.590 | 2.417 | 0.534-10.929 | 3.197 | 0.915-11.187 | 1.088 | 0.477-2.484 | 0.754 | 0.341-1.667 |
| *Length of professional experience* | |  |  |  |  |  |  |  |  |  |  |  |
| <10 years | 0.933 | 0.348-2.514 | 1.487 | 0.811-2.727 | 1.869 | 0.990-3.530 | **1.936** | **1.110-3.378** | 1.378 | 0846-2.246 | **1.883** | **1.163-3.049** |
| 10 to 18 years | ref | | ref | | ref | | ref | | ref | | ref | |
| 19 years and more | 0.657 | 0.277-1.561 | 1.552 | 0.880-2.738 | 0.804 | 0.491-1.316 | 1.385 | 0.853-2.249 | 1.003 | 0.648-1.553 | 1.167 | 0.765-1.781 |

Supplementary Table 2b. Factors associated with the infrequent or no use of the programs using univariable logistic regression

MedSW: medical social worker; SW: social worker in the three institutions such as Community general support centers, Social Welfare Council, and local government; OR: odds ratio; CI: confidence intervals.

*Article 44 of the National Health Insurance Act defines reduction/postponement/exemption based on the judgment of the municipality regarding “people insured for special reasons.”

Statistically significant values are shown in bold.

**Supplementary Figure Legends**

Figure 1a: Each factor is assumed to be independently related to the outcome variable.

Figure 1b: The potential causal relationship is considered for each factor in Figure 1a.


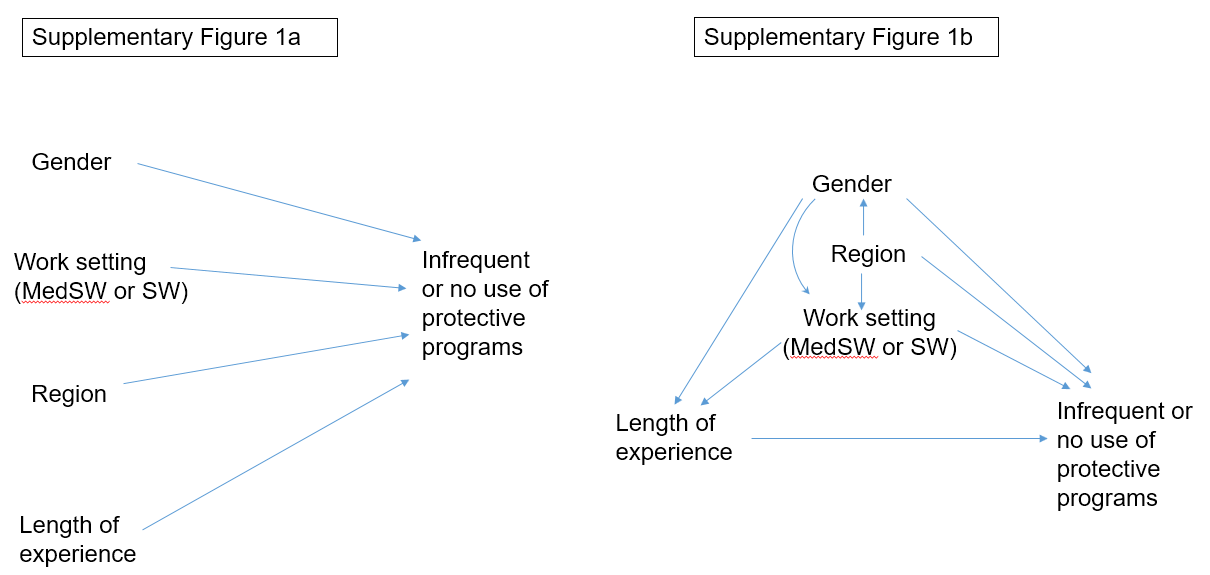

Supplement: Supplementary file 2 — Supplementary file2 (DOCX 82 KB) [file 10615_2023_914_MOESM2_ESM.docx]
